# Brain structure prior to non-central nervous system cancer diagnosis: A population-based cohort study

**DOI:** 10.1016/j.nicl.2020.102466

**Published:** 2020-10-13

**Authors:** Kimberly D. van der Willik, Pinar Yilmaz, Annette Compter, Michael Hauptmann, Katarzyna Jóźwiak, Rikje Ruiter, Bruno H.Ch. Stricker, Meike W. Vernooij, M. Arfan Ikram, Michiel B. de Ruiter, Sanne B. Schagen

**Affiliations:** a Department of Epidemiology, Erasmus MC - University Medical Center Rotterdam, Rotterdam, the Netherlands; b Department of Psychosocial Research and Epidemiology, Netherlands Cancer Institute, Amsterdam, the Netherlands; c Department of Radiology and Nuclear Medicine, Erasmus MC - University Medical Center Rotterdam, Rotterdam, the Netherlands; d Department of Neuro-oncology, Netherlands Cancer Institute, Amsterdam, the Netherlands; e Institute of Biostatistics and Registry Research, Brandenburg Medical School Theodor Fontane, Neuruppin, Germany; f Brain and Cognition, Department of Psychology, University of Amsterdam, Amsterdam, the Netherlands

**Keywords:** Cancer, Brain imaging, Cognitive function, Cohort studies, Epidemiology

## Abstract

**Purpose:**

Many studies have shown that patients with non-central nervous system (CNS) cancer can have brain abnormalities, such as reduced gray matter volume and cerebral microbleeds. These abnormalities can sometimes be present even before start of treatment, suggesting a potential detrimental effect of non-CNS cancer itself on the brain. In these previous studies, psychological factors associated with a cancer diagnosis and selection bias may have influenced results. To overcome these limitations, we investigated brain structure with magnetic resonance imaging (MRI) prior to cancer diagnosis.

**Patients and methods:**

Between 2005 and 2014, 4,622 participants from the prospective population-based Rotterdam Study who were free of cancer, dementia, and stroke, underwent brain MRI and were subsequently followed for incident cancer until January 1st, 2015. We investigated the association between brain MRI measurements, including cerebral small vessel disease, volumes of global brain tissue, lobes, and subcortical structures, and global white matter microstructure, and the risk of non-CNS cancer using Cox proportional hazards models. Age was used as time scale. Models were corrected for e.g. sex, intracranial volume, educational level, body mass index, hypertension, diabetes mellitus, smoking status, alcohol use, and depression sum-score.

**Results:**

During a median (interquartile range) follow-up of 7.0 years (4.9–8.1), 353 participants were diagnosed with non-CNS cancer. Results indicated that persons who develop cancer do not have more brain abnormalities before clinical manifestation of the disease than persons who remain free of cancer. The largest effect estimates were found for the relation between presence of lacunar infarcts and the risk of cancer (hazard ratio [HR] 95% confidence interval [CI] = 1.39 [0.97–1.98]) and for total brain volume (HR [95%CI] per standard deviation increase in total brain volume = 0.76 [0.55–1.04]).

**Conclusion:**

We did not observe associations between small vessel disease, brain tissue volumes, and global white matter microstructure, and subsequent cancer risk in an unselected population. These findings deviate from previous studies indicating brain abnormalities among patients shortly after cancer diagnosis.

## Highlights

• In a population-based setting we studied brain structure before cancer diagnosis.
• Brain structure was not altered before non-CNS cancer diagnosis.
• The effect of cancer on the brain before clinical manifestation is not supported.

## 1 Introduction

Patients with non-central nervous system (CNS) cancer frequently report cognitive problems during and after cancer treatment. (Ahles et al., 2012, Koppelmans et al., 2012, Janelsins et al., 2014) Whereas most research has focused on the effects of cancer treatment (e.g. chemotherapy) on brain health and cognitive function, several studies have shown that cancer patients can have impaired cognitive function even before start of cancer treatment. (Scherling et al., 2012, Menning et al., 2015, Kesler et al., 2017, Hermelink et al., 2007, Patel et al., 2015, Sinha et al., 2018, Bernstein et al., 2018, Hshieh et al., 2018, Simo et al., 2015) This pretreatment cognitive impairment can sometimes persist after adjustment for psychological factors, suggesting that non-CNS cancer may impact the brain apart from cancer treatment, for instance through inflammatory or vascular processes. (Ahles et al., 2012, Patel et al., 2015, Lyon et al., 2016, van der Willik et al., 2018, Olson and Marks, 2019) This hypothesis has further been supported by preclinical studies showing that tumor-bearing, treatment-naïve rodents can have impaired memory function. (Pyter et al., 2010, Yang et al., 2014, Walker et al., 2018)

Understanding the underlying causes of cognitive impairment in non-CNS cancer patients is pivotal to develop prevention and intervention strategies. Several neuroimaging studies have performed brain magnetic resonance imaging (MRI) to investigate the neural underpinnings of cognitive impairment in cancer patients from pre- to posttreatment. (McDonald and Saykin, 2013, Li and Caeyenberghs, 2018, Lange et al., 2019) These studies have shown subtle changes in gray and white matter volumes and frontal lobe hyperactivation before start of treatment, and various brain abnormalities after treatment, including reductions in gray matter volume, cerebral microbleeds, and decreased white matter microstructure. (Simo et al., 2015, Li and Caeyenberghs, 2018, Menning et al., 2015, Menning et al., 2017, Menning et al., 2018, Kesler et al., 2017, Hermelink et al., 2007, Koppelmans et al., 2012, Koppelmans et al., 2015, Lepage et al., 2014, McDonald et al., 2012, Mzayek et al., 2020)

However, these studies are challenged by the effects of psychological factors accompanying a cancer diagnosis, including stress, depression, and anxiety, which may influence brain structure. (Yoshii et al., 2017, Cwik et al., 2020) Also, the feasibility of a baseline assessment after diagnosis but before subsequent treatment is limited, resulting in high rates of non-participation and selection bias. (Jenkins et al., 2016) These limitations can be overcome by studying brain structure and function of cancer patients before cancer diagnosis, with the underlying assumption that cancer is already present, yet not diagnosed.

Within the unique setting of the prospective population-based Rotterdam Study, we have previously shown that the trajectory of cognitive function prior to cancer diagnosis did not differ between participants who developed cancer and those who remained cancer-free during follow-up. (van der Willik et al., 2020) Since in general, changes in brain structure correlate only moderately with cognitive function, (Takeuchi et al., 2017) absence of accelerated change in cognitive function before cancer diagnosis does not preclude presence of abnormalities in brain structure.

Here, we studied the association between brain MRI measurements of cerebral small vessel disease, brain tissue volumes, and white matter microstructure prior to the clinical manifestation of cancer, and the subsequent risk of different types of non-CNS cancer. Such associations may reflect whether there are differences in brain structure between participants who are diagnosed with cancer during follow-up and those who remain cancer-free. This study population is defined by the availability of brain MRI scans. Therefore, the study is conducted in a slightly different sample than the sample in which we found no indication of impaired cognitive function before cancer diagnosis. (van der Willik et al., 2020) For this reason, we also explored the association between cognitive function (self-reported and tested) and the risk of cancer in the current sample.

## 2 Methods

### 2.1 Setting

This study was embedded in the Rotterdam Study, an ongoing population-based prospective cohort study that investigates determinants and occurrence of chronic diseases in the middle-aged and elderly population. The design of the Rotterdam Study has been described in detail previously. (Ikram et al., 2020) Briefly, the initial cohort started in 1990 with 7,983 participants aged ≥ 55 years residing in the district Ommoord in Rotterdam, the Netherlands. The cohort was expanded with 3,011 participants in 2000, followed by an additional inclusion of 3,392 participants aged ≥ 45 years in 2006. From 2005 onwards, brain MRI was implemented into the study protocol of the Rotterdam Study. (Ikram et al., 2015)

Participants were interviewed at home by a trained research assistant, followed by two visits to the research facility for different examinations including laboratory assessments and imaging. Follow-up examinations take place every three to five years.

The Rotterdam Study has been approved by the Medical Ethics Committee of the Erasmus Medical Center and by the Ministry of Health, Welfare and Sport of the Netherlands. Written informed consent was obtained from all participants.

### 2.2 Study population

**Fig. 1:**
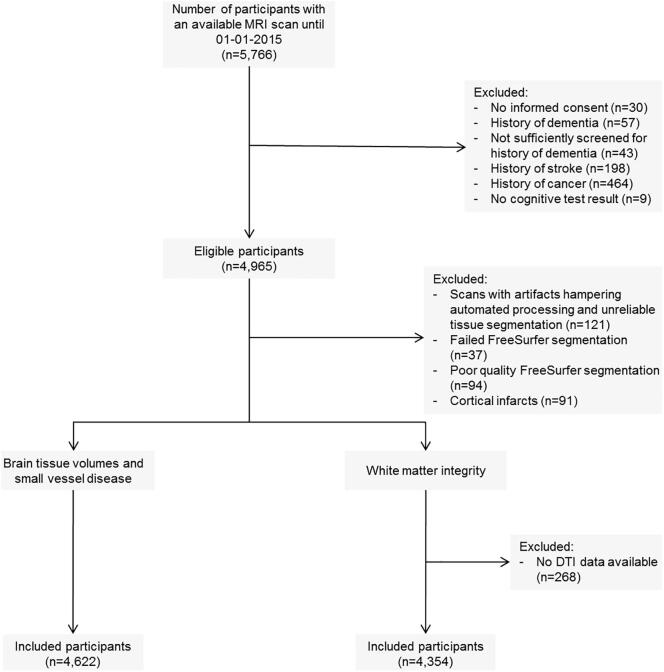
Flowchart of study population. DTI, diffusion tensor imaging; MRI, magnetic resonance imaging.

Out of the 14,926 participants of the Rotterdam Study, 5,766 had at least one brain MRI scan acquired between 2005 and 2014. Of the 5,766 participants, we excluded those without informed consent to access medical files during follow-up (n = 30), with a history of dementia (n = 57) or who were not sufficiently screened for history of dementia (n = 43), with a history of stroke (n = 198), with a history of cancer (n = 464), and those without any cognitive test result (n = 9), resulting in 4,965 eligible participants. Subsequently, we excluded participants who had MRI scans with artifacts and unreliable tissue segmentation (n = 121), without FreeSurfer segmentation (n = 37), with poor FreeSurfer segmentation quality (n = 94) and with MRI-defined cortical infarcts (n = 91), ending up with 4,622 participants. For diffusion tensor imaging (DTI) analyses we additionally excluded participants who had MRI scans, but no available DTI data (n = 268), resulting in 4,354 participants for DTI analyses (Fig. 1). If a participant had multiple MRI scans, we included only the first obtained scan for analyses to avoid bias because of the prospective cohort design.

### 2.3 MRI acquisition and processing

Brain MRI was performed on a 1.5-tesla MRI scanner with a dedicated 8-channel head coil (General Electric Healthcare, Milwaukee, USA). The scan protocol and sequence details have been described in detail previously and are summarized in Supplementary Table 1. (Ikram et al., 2015) Scans for brain volumetry included T1-weighted (voxel size 0.49 × 0.49 × 1.6 mm^3^), proton density-weighted (voxel size 0.6 × 0.98 × 1.6 mm^3^), and fluid-attenuated inversion recovery (FLAIR, voxel size 0.78 × 1.12 × 2.5 mm^3^) sequences which were used for automated segmentation of supratentorial gray matter volume, white matter volume, cerebrospinal fluid, and white matter hyperintensities. (de Boer et al., 2009, Vrooman et al., 2007) Pre-processing included co-registration, correction of non-uniformity, and variance scaling. Before segmentations, the brain is extracted from the scan using a manually segmented brain mask that is non-rigidly registered to the T1-weighted image using Elastix. (Klein et al., 2010) We used the k-nearest neighbor segmentation to classify scans into brain tissue volumes and cerebrospinal fluid. (Anbeek et al., 2005) All segmentations were inspected and manually corrected if necessary using a dedicated tool that has been developed in MevisLab that can visualize the original scan with the image processing results. (Ikram et al., 2015) Editing tools were available to adjust segmentations if necessary. Manual editing of errors was needed in less than ten percent.

Markers of cerebral small vessel disease included white matter hyperintensity volume (mL), presence of cerebral microbleeds, and presence of infarcts. Cerebral microbleeds were rated on a 3-dimensional, T2*-weighted gradient-recalled echo MRI scan (voxel size 0.78 × 1.12 × 1.6 mm^3^) as focal areas of very low signal intensity. Infarcts were categorized as cortical infarcts (infarcts with involvement of cortical gray matter which were excluded for analyses for reliability of tissue segmentations) and lacunar infarcts (focal lesions between 3 and 15 mm in non-cortical tissue with signal intensity similar to that of cerebrospinal fluid on all sequences, and, when located supratentorially, with a hyperintense rim on the FLAIR sequence). (Vernooij et al., 2007, Vernooij et al., 2008)

Total brain volume (mL) was defined as the sum of gray matter volume (mL) and white matter volume (mL, sum of normal appearing white matter and white matter hyperintensity volume). Intracranial volume (mL) was the sum of total brain volume and cerebrospinal fluid. Although these volumes were restricted to the supratentorial region, we refer to these volumes as total brain volume and intracranial volume. Lobar volumes were segmented by using an atlas in which the lobes were manually outlined. (Bokde et al., 2005) This atlas was subsequently non-rigidly transformed to each brain to obtain the volume of each lobe. (Ikram et al., 2015) Lobar volumes included both gray matter and white matter. T1-weighted MR images were processed using FreeSurfer (version 6.0) to calculate cortical thickness (mm), cortical surface area (mm^2^), and subcortical volumes (mL) of the hippocampus, amygdala, caudate, putamen, thalamus, and pallidum (FreeSurfer is freely available for download online at http://surfer.nmr.mgh.harvard.edu/). For quality assessment, we have randomly selected a subset of scans that we visually inspected. Next, we identified a cut-off on our automated quality assessment metric which allowed us to exclude unusable FreeSurfer data. (White et al., 2018) This cut-off has subsequently been applied to the remaining data and all scans below this cut-off were excluded. We have confirmed that several metrics (e.g., cortical thickness) have no significant correlation with the automated quality assessment metric after the exclusions have been performed. (Lamballais et al., 2020).

Measurements of white matter microstructure were obtained from DTI (supratentorially, voxel size 3.3 × 2.2 × 3.5 mm^3^), which was embedded in the protocol of the Rotterdam Study from March 2006 onwards. (Vrooman et al., 2007, Koppelmans et al., 2014) Echo-planar imaging (EPI) was used as readout module. Normal appearing white matter was distinguished from white matter hyperintensities using an automatic post-processing step based on the FLAIR image and the tissue segmentation. (de Boer et al., 2009) Next, the segmentation of white matter hyperintensities was mapped into DTI image space using boundary-based registration performed on the white matter segmentation and the T1-weighted image. (Greve and Fischl, 2009) Co-registrations of the DTI to the T1-weighted image were visually inspected to ensure a good fit and that DTI measures did not include gray matter or cerebrospinal fluid partial volumes. This co-registration partly corrected potential non-linear changes induced by the EPI readout module. DTI data were pre-processed using a standardized pipeline that included correction for subject motion and Eddy currents, estimation of the diffusion tensor, and registration to tissue segmentation matter. (Koppelmans et al., 2014) Diffusion tensors were estimated using a non-linear Levenberg-Marquardt estimator (available in Explore DTI), (Leemans et al.,2009) from which global mean fractional anisotropy (FA) and mean diffusivity (MD, 10^−3^ mm^2^/s) in the normal appearing white matter were obtained. FA reflects the degree of diffusion directionality of water molecules. (Alexander et al., 2007) MD represents the average diffusion of water molecules. Lower FA and higher MD are indications of lower white matter microstructure. DTI images were manually inspected for registration and segmentation and corrected where possible. Between February 2007 and May 2008, 1,169 participants were scanned with the phase and frequency encoding directions swapped for the diffusion acquisition due to a technical issue. We have therefore included phase encoding direction as covariate in the analyses (see statistical analysis). (de Groot et al., 2015)

### 2.4 Cognitive function assessment

Cognitive function was assessed by a neuropsychological test battery administered at the research center. Assessments took place between 2002 and 2014. The cognitive assessment corresponding to the same visit round as the visit round of the MRI scan was used, with a median (interquartile range [IQR]) time between cognitive function assessment and MRI scan of −0.13 years (-0.31 to −0.08). Up to 2015, the following cognitive tests were administered: Mini-Mental State Examination (MMSE), Word Fluency Test (WFT), Letter-Digit Substitution Test (LDST), Stroop Test (reading, naming, interference), Purdue Pegboard Test (PPT, right, left, both hands), and 15-Word Learning Test (WLT, immediate recall, delayed recall, recognition). (Folstein et al., 1975, Houx et al., 1993, van der Elst et al., 2006, Tiffin and Asher, 1948, Van der Elst et al., 2006, Bleecker et al., 1988)

Global cognitive function was assessed by the general cognitive factor based on WFT, LDST, Stroop Test: interference, sum-score of individual PPTs, and WLT: delayed recall and was identified as the first unrotated component of a principal component analysis, which explained at least 48.0% of the total variance in individual cognitive tests. (Hoogendam et al., 2014) The general cognitive factor was only computed if all five individual tests were completed.

Self-reported memory complaints were measured with three yes/no questions: 1) Do you have more problems remembering things than before? 2) Has there been an increase in the times that you forgot what you were up to? 3) Do you have more word-finding problems than before?

### 2.5 Ascertainment of cancer

Diagnoses of cancer were based on medical records of general practitioners (including hospital discharge letters) and through linkage with Dutch Hospital Data, Netherlands Cancer Registry, and histology and cytopathology registries in the region. (Ikram et al., 2020) Incident cancer was defined as any primary malignant tumor, excluding non-melanoma skin cancer. Diagnoses were coded independently by two physicians according to the International Classification of Diseases, tenth revision (ICD-10). In case of discrepancy, consensus was sought through consultation with a physician specialized in internal medicine. Date of diagnosis was based on date of biopsy (solid tumors) and laboratory assessment (hematologic tumors), or – if unavailable – date of hospital admission or discharge letter. Only pathology-confirmed cancers were included in the analysis. Follow-up of cancer registration was completed up to January 1st, 2015.

### 2.6 Measurement of covariates

During home interviews, participants provided information on educational level, smoking status, and alcohol use. Educational level was classified into primary, lower (lower or intermediate general education, or lower vocational education), intermediate (intermediate vocational education or higher general education), or higher (higher vocational education or university). Smoking was categorized as never, current, or former. Alcohol use was classified into any use or no use of alcohol. At the research center, height and weight were measured from which the body mass index (BMI, kg/m^2^) was computed. Furthermore, systolic and diastolic blood pressures were measured twice on the right arm with a random-zero sphygmomanometer of which the mean was used for analyses. Hypertension was defined as a systolic blood pressure of ≥ 140 mm Hg, a diastolic blood pressure of ≥ 90 mm Hg, or use of antihypertensive medication. (Engberink et al., 2012) Diabetes mellitus was defined as fasting serum glucose level ≥ 7.1 mmol/L, a random serum glucose level ≥ 11.1 mmol/L, or use of glucose-lowering medication. ([58]) Symptoms of depression were evaluated with the Center for Epidemiologic Studies Depression scale (CES-D), which was converted to a sum-score. (Mirza et al., 2014)

### 2.7 Statistical analysis

We investigated the association between brain MRI measurements including cerebral small vessel disease, brain tissue volumes, and white matter microstructure, and the risk of cancer using Cox proportional hazards models. (Cox and Oakes, 1984) Cox proportional hazards models are semiparametric regression models for survival data and can be used to obtain hazard ratios (HRs) and 95% confidence intervals (95%CIs). The hazard is the instantaneous risk of an event at time *t*, given that the event has not occurred until time *t*. In the current study, we are interested in cancer as the event. For interpretation purposes and to facilitate comparisons across different MRI measurements, we standardized continuous brain MRI measurements (i.e., white matter hyperintensity volume, brain tissue volumes, and white matter microstructure) by creating Z-scores (individual value minus population mean, divided by population standard deviation [SD]). Therefore, the HR for continuous variables indicates the change in the risk of cancer if the brain MRI measurement of interest rises by one SD. (Zwiener et al., 2011) A HR above one indicates that the risk of cancer increases for every SD increase in the brain MRI measurement. For categorical variables (i.e., cerebral microbleeds and lacunar infarcts) the hazard ratio can be interpreted as the ratio of the hazard for cancer at time *t* for participants with microbleeds or infarcts to the hazard for cancer at *t* for those without microbleeds or infarcts. A HR above one indicates that participants with microbleeds or infarcts have a higher risk of cancer than participants without microbleeds or infarcts.

White matter hyperintensity volume was transformed using the natural logarithm to reach a normal distribution. For volumes of the lobes and subcortical structures we used the average of the left and right hemisphere. We explored non-linear associations by categorizing global brain volumes into quantiles. For each MRI measurement, we constructed two nested models. Covariates were selected based on previous literature (VanderWeele, 2019) on the relation between cancer, brain abnormalities, and cognitive function. In Model I, the effect of each MRI measurement was adjusted for sex and intracranial volume. In a middle-aged to elderly population, correcting for intracranial volume is preferred over correcting for total brain volume to better estimate the extent of global atrophy or atrophy between different regions. (O'Brien et al., 2011, Voevodskaya et al., 2014) In addition to these adjustments for all MRI measurements, the effect of gray matter volume was adjusted for total white matter volume (i.e., normal appearing white matter volume plus white matter hyperintensity volume), and analyses for measurements of white matter microstructure were adjusted for normal appearing white matter volume, white matter hyperintensity volume, and phase encoding direction. Model II was Model I plus additional adjustment for educational level (primary, lower, intermediate, higher), BMI (continuous), hypertension (yes, no), diabetes mellitus (yes, no), smoking status (never, current, former), alcohol use (yes, no), and CES-D sum-score (continuous). An overview of the distributions of the continuous determinants and covariates used in the models is provided in Supplementary Fig. 1. (Allen et al., 2019) Ethnicity was not used as a covariate since nearly all participants (97.0%) were of European descent. Age was used as the underlying time scale in all Cox models to control for the confounding effects of age and to allow a non-parametric age effect. (Lamarca et al., 1998, Canchola et al., 2003) Follow-up time was measured from the date of first MRI scan until the date of cancer diagnosis, death, loss to follow-up, or January 1st, 2015, whichever came first. Participants with CNS cancer were censored at date of diagnosis (i.e., follow-up was terminated at date of CNS cancer diagnosis), because mechanisms underlying brain abnormalities differ between non-CNS and CNS cancers, given that CNS cancer can cause direct damage to the brain. (Coomans et al., 2019) Multicollinearity was checked by calculating the Variance Inflation Factor (VIF). None of the covariates had a VIF above ten. (James et al., 2013) The proportional hazards assumption was checked by visual inspection of the Schoenfeld residuals. (Schoenfeld, 1982)

Given that cortical gray matter volume is approximated by the product of cortical thickness and cortical surface area, we explored whether any association between gray matter volume and risk of cancer may be driven by one of these features. Cortical surface area is the main determinant of variation in cortical gray matter volumes between individuals. (Storsve et al., 2014) Cortical thickness and surface area decrease both during aging, but it has been shown that reduced cortical thickness is probably the main driver of decreasing cortical gray matter volume.

Subsequently, to investigate the robustness of our findings, we conducted sensitivity analyses in which we limited the analyses to a shorter follow-up time by censoring all participants two years after the MRI scan. Cancer might indirectly affect the brain through inflammatory or vascular processes. (Olson and Marks, 2019) Tumor progression has been associated with inflammation and vascular changes. (Coussens and Werb, 2002) We therefore hypothesized that if growing, yet undiagnosed cancer affects the brain, brain abnormalities will become more apparent closer to the date of cancer diagnosis.

Next, we analyzed effects separately for the most frequent cancer types (breast, prostate, colorectal, lung) and cancer stage (local versus metastasized). In addition, we studied effect modification for sex by stratifying. We adjusted these models for the same covariates that were used in Model II. Participants were censored at time of cancer diagnosis if they were diagnosed with another type of cancer than the cancer type of interest.

We subsequently investigated the relation between tested cognitive function and self-reported memory function, and the risk of cancer. A Cox model with a particular cognitive test result included also information on sex, educational level, BMI, hypertension, diabetes mellitus, smoking status, alcohol use, and CES-D sum score. The cognitive test results were standardized by creating Z-scores to facilitate comparisons across the different measures.

Lastly, we repeated analyses using the MRI scan closest to cancer diagnosis in a matched cohort design by matching each participant with cancer to three cancer-free participants based on age, sex, and follow-up time. These analyses provided similar findings to those obtained from the original cohort design using the first available MRI scan and are therefore not reported separately.

Multiple imputation was used for missing covariates (maximum of 0.9%) based on determinants, outcome, and covariates. The missing values were imputed five times, resulting in five datasets. Rubin’s method was used to estimate pooled HRs and 95%CIs from these five datasets. (Rubin, 1987) A two-sided *P*-value of < 0.05 was considered statistically significant. We did not correct for multiple testing, because the brain MRI measurements were not independent from each other and the analyses were exploratory. Correction for multiple testing may therefore be too conservative. (Gelman et al., 2012) In total, 36 Cox proportional hazards models were run for the main analyses, six to explore non-linear associations by categorizing global brain tissue volumes, 18 for analyses stratified by sex, 90 for analyses stratified by cancer type, 18 for sensitivity analyses, and 14 for analyses on cognitive function. All analyses were performed using the ‘survival’ package from R software Version 3.4.1. (Therneau, 2005)

## 3 Results

Characteristics of participants at time of MRI scan are presented in Table 1. During a median (IQR) follow-up of 7.0 years (4.9–8.1), 353 out of 4,622 participants (7.6%) were diagnosed with cancer. The most frequently diagnosed cancer types were prostate (16.1%), female breast (13.0%), colorectal (17.8%), and lung (10.5%). The median time (IQR) between MRI scan and cancer diagnosis was 3.3 years (1.7–5.6), with a mean (SD) age of 70.6 years (9.0) at diagnosis.

**Table 1:** Baseline characteristics of total study population.

**Characteristic**	**All participants(N = 4,622)**
Age^a^	61.6 years (55.5–71.7)
Sex	
Women	2,574 (55.7)
Men	2,048 (44.3)
Education	
Primary	390 (8.4)
Lower	1,743 (37.7)
Intermediate	1,368 (29.6)
Higher	1,080 (23.4)
Body mass index^b^	27.4 kg/m^2^ (4.1)
Hypertension	
No	1,776 (38.4)
Yes	2,823 (61.1)
Diabetes mellitus	
No	4,292 (92.9)
Yes	315 (6.8)
Smoking	
Never	1,426 (30.9)
Former	2,436 (52.7)
Current	734 (15.9)
Alcohol use	
No	525 (11.4)
Yes	4,072 (88.1)
CES-D sum score^a^	4.0 (3.0–8.0)
Cerebral small vessel disease	
White matter hyperintensity volume^a^	2.8 mL (1.6–5.7)
Microbleeds	840 (18.2)
Lacunar infarcts	283 (6.1)
Global brain tissue volume^b^	
Intracranial volume	1,138.9 mL (116.1)
Total brain volume	939.9 mL (100.6)
Gray matter	530.6 mL (55.4)
Normal appearing white matter	403.8 mL (60.9)
Lobar brain tissue volume^b^	
Frontal	79.5 mL (8.2)
Parietal	52.0 mL (5.6)
Temporal	49.1 mL (5.3)
Occipital	22.8 mL (2.8)
Subcortical structure volume^b^	
Hippocampus	3.9 mL (0.4)
Amygdala	1.4 mL (0.2)
Caudate	3.3 mL (0.5)
Putamen	4.2 mL (0.5)
Thalamus	6.6 mL (0.7)
Pallidum	1.6 mL (0.2)
White matter microstructure^b,c^	
Global fractional anisotropy	0.34 (0.02)
Global mean diffusivity	0.74 * 10^-3^ mm^2^/s (0.03)
Cognitive functiona,^d^	
Mini-Mental State Examination	28.0 (27.0–29.0)
Word Fluency Test^b^	23.0 (5.9)
Letter-Digit Substitution Test^b^	30.6 (6.9)
Stroop Test: naming	16.4 (14.7–18.5)
Stroop Test: reading	22.4 (20.0–25.4)
Stroop Test: interference	44.3 (37.2–54.3)
Purdue Pegboard Test^b^	36.2 (5.2)
Word Learning Test: immediate recall^b^	7.8 (2.1)
Word Learning Test: delayed recall^b^	7.8 (2.9)
Word Learning Test: recognition	14.0 (13.0–15.0)
General cognitive factor^b^	0.0 (1.0)
Self-reported memory complaints^e^	
More problems remembering	2,082 (46.4)
Forgetting (daily) pursuits	1,318 (29.4)
Word-finding problems	1,182 (26.3)

Data are presented as number (percentage) of participants unless otherwise indicated.

Values are shown without imputation and therefore not always add up to 100%.

CES-D, Center for Epidemiological Studies Depression Scale; N, number of participants.

a Presented as median (interquartile range). ^b^ Presented as mean (standard deviation). ^c^ FA and MD were measured in 4,354 participants due to missing diffusion tensor imaging data. ^d^ Number of participants differed per cognitive test. ^e^ Self-reported memory complaints were measured in 4,486 participants.

### 3.1 Cerebral small vessel disease

No associations were found between white matter hyperintensity volume or presence of microbleeds and the risk of cancer (HR [95%CI] per SD increase in white matter hyperintensity volume = 0.98 [0.87–1.09], *P* = .67 and for presence of microbleeds = 1.00 [0.77–1.29], *P* = .98, Table 2). The largest HR for cerebral small vessel disease was observed for presence of lacunar infarcts and the risk of all cancers combined (HR [95%CI] = 1.39 [0.97–1.98], *P* = .07, Table 2). This effect estimate was more pronounced in sensitivity analyses when censoring the follow-up time after the first two years after the MRI scan (HR [95%CI] = 1.65 [0.95–2.86], *P* = .07, Supplementary Table 2).

**Table 2:** Association between markers of cerebral small vessel disease and risk of cancer.

**MRI measurement**	**Cancer(n/N = 353/4,622)**			
	Model IHR (95% CI)	*P*-value	Model IIHR (95% CI)	*P*-value
White matter hyperintensity volume, mLa,^b^	0.99 (0.88–1.10)	0.81	0.98 (0.87–1.09)	0.67
Microbleeds	1.01 (0.78–1.31)	0.96	1.00 (0.77–1.29)	0.98
Lacunar infarcts	1.46 (1.02–2.07)	0.04	1.39 (0.97–1.98)	0.07

Model I: adjusted for sex and total intracranial volume. Model II: model I plus adjusted for education, body mass index, hypertension, diabetes mellitus, smoking status, alcohol use, and CES-D sum score.

CES-D, Center for Epidemiological Studies Depression Scale; CI, confidence interval; HR, hazard ratio; MRI, magnetic resonance imaging; n, number of participants with incident cancer; N, number of participants.

a Expressed per standard deviation increase. ^b^ Transformed with a natural logarithm.

We found no differences in associations for different cancer types (Supplementary Table 4), nor between men and women (Supplementary Table 6).

### 3.2 Brain tissue volumes

Overall, we found no associations between global and lobar brain tissue volumes and the risk of cancer. The majority of the effect estimates for brain tissue volumes were below one, with the most pronounced HR for total brain volume and the risk of cancer (HR [95%CI] per SD increase in total brain volume = 0.76 [0.55–1.04], *P* = .09, Table 3). We did not observe a non-linear pattern when categorizing the volumes into quantiles (data not shown). No associations were found between cortical thickness and risk of cancer (HR [95%CI] per SD increase in cortical thickness = 0.94 [0.84–1.06], *P* = .33), and cortical surface area and risk of cancer (HR [95%CI] per SD increase in cortical surface area = 0.93 [0.72–1.20], *P* = .58). Regarding subcortical structures, the most pronounced effect estimate was found for hippocampal volume and the risk of cancer (HR [95%CI] = 0.87 [0.75–1.01], *P* = .07, Table 3).

**Table 3:** Association between brain tissue volumes and microstructural brain measurements and risk of cancer.

**MRI measurement**^a^	**Cancer(n/N = 353/4,622)**			
	Model IHR (95% CI)	*P*-value	Model IIHR (95% CI)	*P*-value
*Global brain tissue volume, mL*				
Total brain volume	0.74 (0.54–1.01)	0.06	0.76 (0.55–1.04)	0.09
Gray matter	0.89 (0.71–1.11)	0.31	0.91 (0.73–1.14)	0.41
Normal appearing white matter	0.86 (0.73–1.02)	0.09	0.87 (0.73–1.03)	0.11
*Lobar brain tissue volume, mL*				
Frontal	0.87 (0.70–1.08)	0.22	0.90 (0.73–1.12)	0.34
Parietal	0.86 (0.71–1.05)	0.15	0.87 (0.72–1.07)	0.19
Temporal	0.90 (0.74–1.10)	0.32	0.92 (0.75–1.13)	0.43
Occipital	0.99 (0.85–1.14)	0.84	0.99 (0.85–1.14)	0.85
*Subcortical structure volume, mL*				
Hippocampus	0.86 (0.74–1.00)	0.05	0.87 (0.75–1.01)	0.07
Amygdala	0.99 (0.86–1.15)	0.94	1.00 (0.86–1.15)	0.95
Caudate	1.04 (0.92–1.17)	0.55	1.03 (0.92–1.16)	0.61
Putamen	0.91 (0.79–1.03)	0.15	0.90 (0.79–1.03)	0.13
Thalamus	0.94 (0.80–1.12)	0.51	0.95 (0.80–1.12)	0.52
Pallidum	0.97 (0.85–1.10)	0.63	0.97 (0.85–1.11)	0.68
*White matter microstructure^b^*				
Global fractional anisotropy	0.98 (0.86–1.12)	0.79	0.98 (0.86–1.12)	0.75
Global mean diffusivity,10^-3^ mm^2^/s	1.02 (0.87–1.19)	0.85	1.01 (0.86–1.19)	0.89

Model I: adjusted for sex and total intracranial volume. For gray matter volume additionally adjustment for total white matter volume. For white matter microstructure additional adjustment for normal appearing white matter volume, white matter hyperintensity volume, and phase encoding direction. Model II: model I plus adjusted for education, body mass index, hypertension, diabetes mellitus, smoking status, alcohol use, and CES-D sum score.

CES-D, Center for Epidemiological Studies Depression Scale; CI, confidence interval; HR, hazard ratio; MRI, magnetic resonance imaging; n, number of participants with incident cancer; N, number of participants.

a Expressed per standard deviation increase. ^b^ Fractional anisotropy and mean diffusivity were measured in 4,354 participants due to missing diffusion tensor imaging data.

When limiting the follow-up to two years after the MRI scan, effect estimates were more pronounced for the association between volumes of total brain and hippocampus with the risk of cancer (Supplementary Table 3, HR [95%CI] per SD increase in total brain volume = 0.63 [0.35–1.12], *P* = .12 and per SD increase in hippocampal volume = 0.75 [0.58–0.98], *P* = .04).

**Fig. 2:**
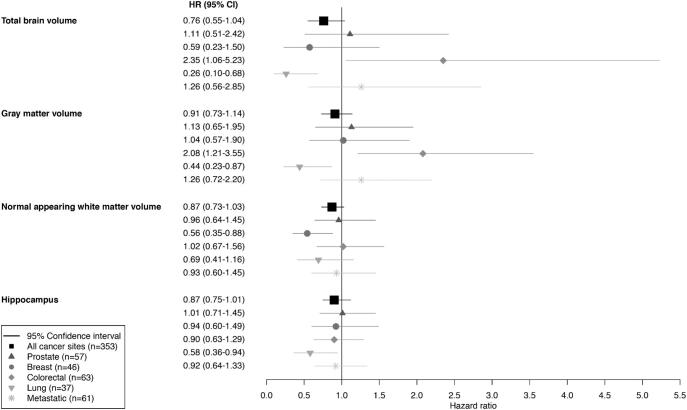
Adjusted hazard ratios for the association between global brain tissue volumes and hippocampus and risk of cancer at different organ sites and metastasized stage. Hazard ratios are expressed per standard deviation increase in volume. Hazard ratios are adjusted for total intracranial volume, sex, education, body mass index, hypertension, diabetes mellitus, smoking status, alcohol use, and CES-D sum score. For gray matter volume additionally adjustment for total white matter volume. The boxes represent the effect size and the horizontal lines indicate the corresponding 95% confidence intervals. CI, confidence interval; HR, hazard ratio.

Regarding cancer type, we found that higher volumes of total brain, gray matter, and hippocampus were associated with a statistically significantly lower risk of lung cancer (Fig. 2). In contrast, higher volumes of total brain and gray matter were associated with a higher risk of colorectal cancer. No differences were observed for the other cancer types and for metastasized cancer, but small numbers led to wide confidence intervals. Results for the remaining brain tissue volumes and risk of cancer stratified by cancer type are shown in Supplementary Table 5.

Lastly, we found no evidence for effect modification by sex (Supplementary Table 7).

### 3.3 White matter microstructure

Global measurements of white matter microstructure were not associated with the risk of cancer (HR [95%CI] per SD increase in global FA = 0.98 [0.86–1.12], *P* = .75 and in global MD = 1.01 [0.86–1.19], *P* = .89, Table 3 and Supplementary Table 3).

Also no associations were found when stratifying by cancer type (Supplementary Table 5) and by sex (Supplementary Table 7).

### 3.4 Cognitive function

All effect estimates for the relation between individual cognitive tests and the risk of cancer were around 1.0, indicating that there are no associations between different cognitive test scores and the risk of cancer (Supplementary Table 8). Per SD increase in the general cognitive factor as measurement of global cognitive function the HR for cancer was 1.03 [0.89–1.20], *P* = .66. Also no associations were found between self-reported memory function and the risk of cancer.

## 4 Discussion

In this population-based study, we aimed to obtain more insight into the impact of cancer on brain structure by investigating the presence of brain abnormalities in non-CNS cancer patients prior to the clinical manifestation of cancer. We found no meaningful associations between cerebral small vessel disease, brain tissue volumes, and white matter microstructure, and the risk of cancer. These findings suggest that persons who develop cancer do not have more brain abnormalities before cancer diagnosis than persons who remain free of cancer.

Our current findings obtained prior to cancer diagnosis deviate from previously observed brain changes after diagnosis but before treatment such as lower gray matter volume and white matter microstructure (i.e., lower FA and higher axial diffusivity). (Scherling et al., 2012, Simo et al., 2015, McDonald et al., 2013) Although we did not find any statistically significant associations, we observed that almost all effect estimates for brain tissues volumes were below one, suggesting that we cannot completely rule out a subtle effect of cancer on the brain. In addition, effect estimates for the association between presence of lacunar infarcts, total brain volume, hippocampal volume, and the risk of cancer were more pronounced when the study follow-up was limited to two years after MRI scan. This may indicate that brain changes (i.e., more lacunar infarcts and smaller brain volumes) become more apparent closer to the date of cancer diagnosis. Given that we did not observe this pattern for any of the cognitive tests, this might suggest that brain changes might arise before they become clinically apparent, as seen in dementia. (Ikram et al., 2010) This may also apply to cancer patients, with cognitive function first being preserved by compensation, followed by loss of compensatory activation, which results eventually in cognitive impairment. (Ahles et al., 2012) Different underlying mechanisms by which non-CNS cancer may affect the brain have been proposed, including peripheral inflammation triggering neurotoxic cytokine response, oxidative stress, or vascular changes (Ahles et al., 2012, Patel et al., 2015, Lyon et al., 2016, van der Willik et al., 2018, Olson and Marks, 2019, Winocur et al., 2017). In addition, the associations were most pronounced for lung cancer, which is strongly associated with inflammation and oxidative stress. (Pine et al., 2011, Filaire et al., 2013) Accordingly, we can conclude that if subclinical non-CNS cancer affects the brain, the effects are limited and may only result in subtle changes that are not evidently detected by measures of supratentorial brain tissue volumes, subcortical brain structure volumes, white matter pathology, and white matter microstructure, or effects are restricted to certain types of non-CNS cancer, such as lung cancer.

Our study has some limitations. First, measurement error in brain MRI volumes might have attenuated the association. For instance, it might have been possible that usage of a higher magnetic field strength or alternative imaging processing pipelines would have resulted in a more pronounced association between certain MRI measurements and the risk of cancer. (Mzayek et al., 2020, Ma et al., 2019) Second, although the statistical power in our main analysis was sufficient to detect a potential association (we were powered to detect a HR of 0.84 for the relation between total brain volume and risk of cancer [α = 0.05, β = 0.80]), the power might have been too limited to find statistically significant associations when limiting the follow-up time to two years and when focusing on different cancer types. Therefore, replication of this study in a larger sample with MRI scans performed more closely to the clinical manifestation of cancer is desirable. (Menning et al., 2015) In addition, it would be interesting to investigate the change in MRI measurements from before to after cancer diagnosis. Third, we had no information on fatigue and frailty, which may be confounding factors that would have further attenuated the effect estimates. Fourth, with the current analyses we were not able to study interrelationships between different brain MRI measurements and therefore we might have missed more complex patterns of brain abnormalities related to the risk of cancer.

Strengths of this study include the unique design by which we could assess brain MRI before clinical manifestation of cancer. Hereby, we excluded the effects of psychological factors associated with a cancer diagnosis on the brain and the potential effects of selection bias. (Yoshii et al., 2017, Cwik et al., 2020) Also, we have a larger sample size than that of other studies assessing brain MRI in cancer patients prior to treatment (number of patients ranging between 10 and 74, compared to 353 patients in our study), and we included different cancer types as outcome whereas previous studies primarily focused on breast cancer.

In conclusion, we found that persons who develop non-CNS cancer did not have more brain abnormalities before cancer diagnosis than persons who remained free of cancer. Our findings do not support that non-CNS cancer affects global brain structure measurements before clinical manifestation of cancer.

## 5 Support

This study was funded by the Dutch Cancer Society (grant number NKI-20157737). Furthermore, the Rotterdam Study is funded by Erasmus Medical Center and Erasmus University, Rotterdam, Netherlands Organization for the Health Research and Development (ZonMw), the Research Institute for Diseases in the Elderly (RIDE), the Ministry of Education, Culture and Science, the Ministry for Health, Welfare and Sports, the European Commission (DG XII), and the Municipality of Rotterdam. The funders had no role in study design, data collection and analysis, decision to publish, or preparation of the manuscript.

## CRediT authorship contribution statement

**Kimberly D. van der Willik:** Conceptualization, Methodology, Formal analysis, Investigation, Data curation, Writing - original draft, Visualization. **Pinar Yilmaz:** Conceptualization, Methodology, Data curation, Writing - original draft. **Annette Compter:** Conceptualization, Writing - review & editing. **Michael Hauptmann:** Methodology, Writing - review & editing. **Katarzyna Jóźwiak:** Methodology, Writing - review & editing. **Rikje Ruiter:** Data curation, Writing - review & editing. **Bruno H.Ch. Stricker:** Data curation, Writing - review & editing. **Meike W. Vernooij:** Conceptualization, Methodology, Writing - review & editing, Supervision. **M. Arfan Ikram:** Conceptualization, Methodology, Writing - review & editing, Supervision. **Michiel B. de Ruiter:** Conceptualization, Methodology, Writing - original draft, Supervision. **Sanne B. Schagen:** Conceptualization, Methodology, Writing - original draft, Supervision, Funding acquisition.

## Declaration of Competing Interest

The authors declare that they have no known competing financial interests or personal relationships that could have appeared to influence the work reported in this paper.
