# Supplementary material for: Brain structure prior to non-central nervous system cancer diagnosis: A population-based cohort study
Source: Neuroimage Clin. 2020 Oct 13;28:102466. doi: 10.1016/j.nicl.2020.102466 (PMC7578754; doi:10.1016/j.nicl.2020.102466)
Supplement: Supplementary data 1 [file mmc1.docx]

**
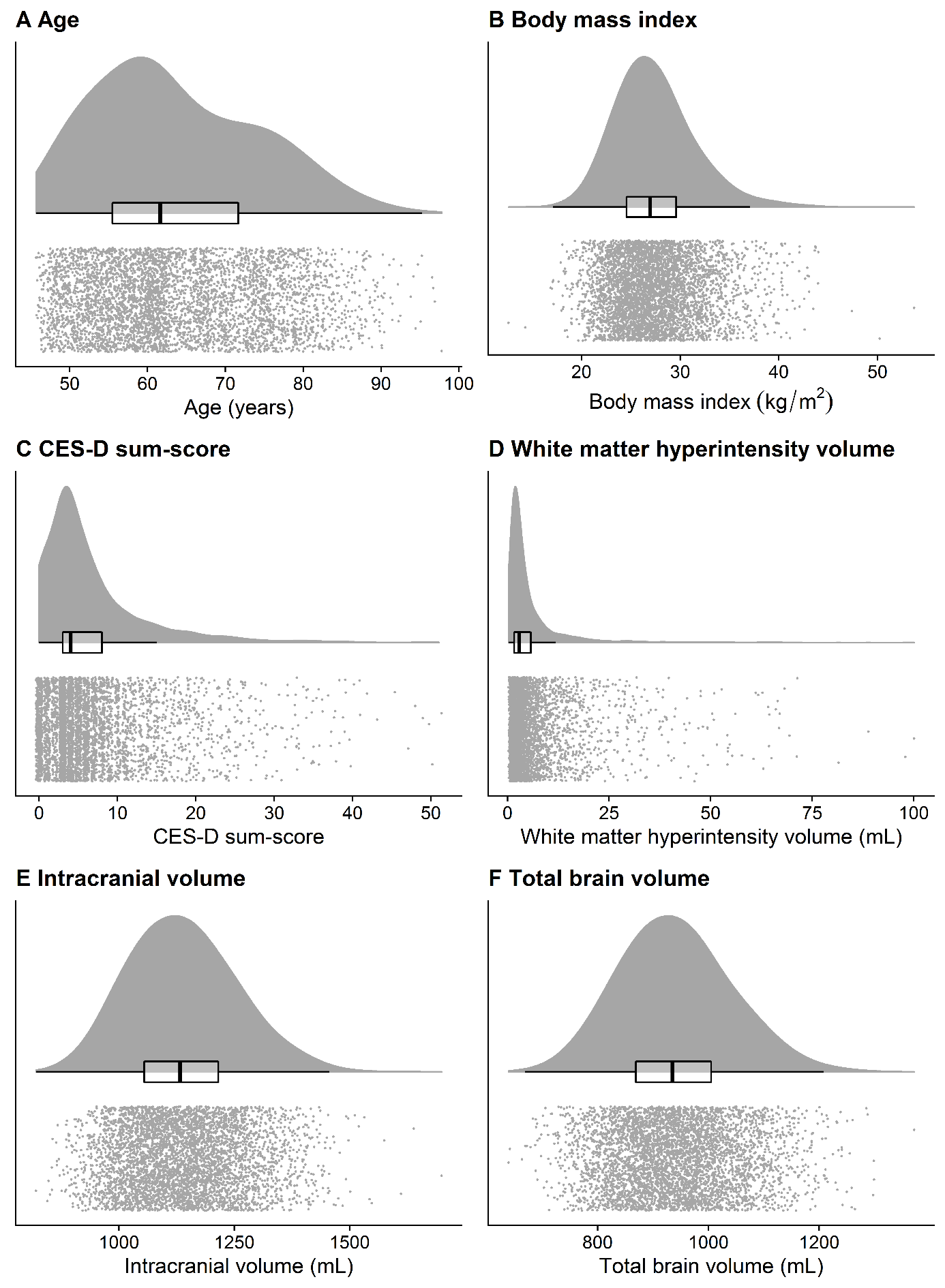
**

**
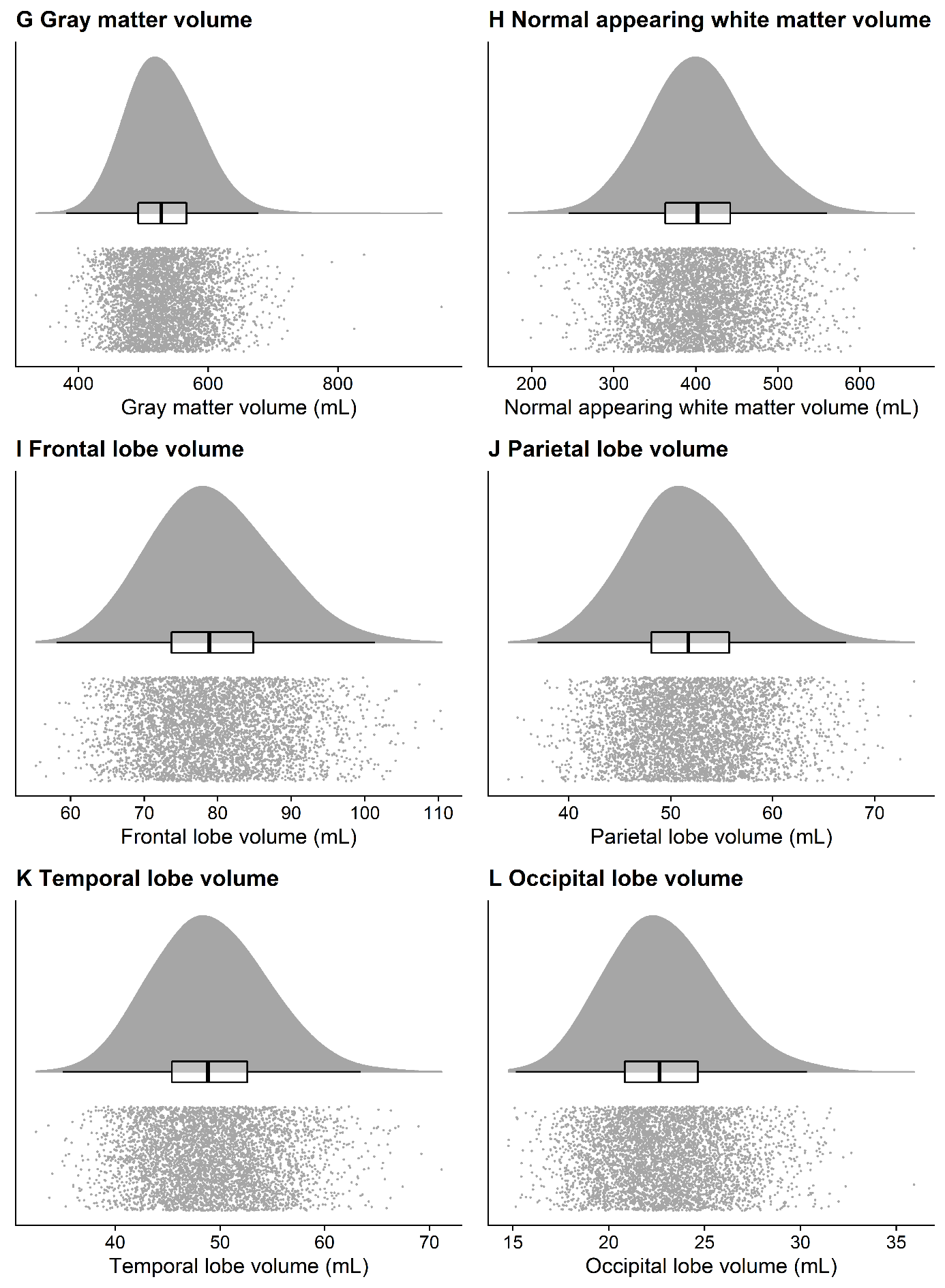

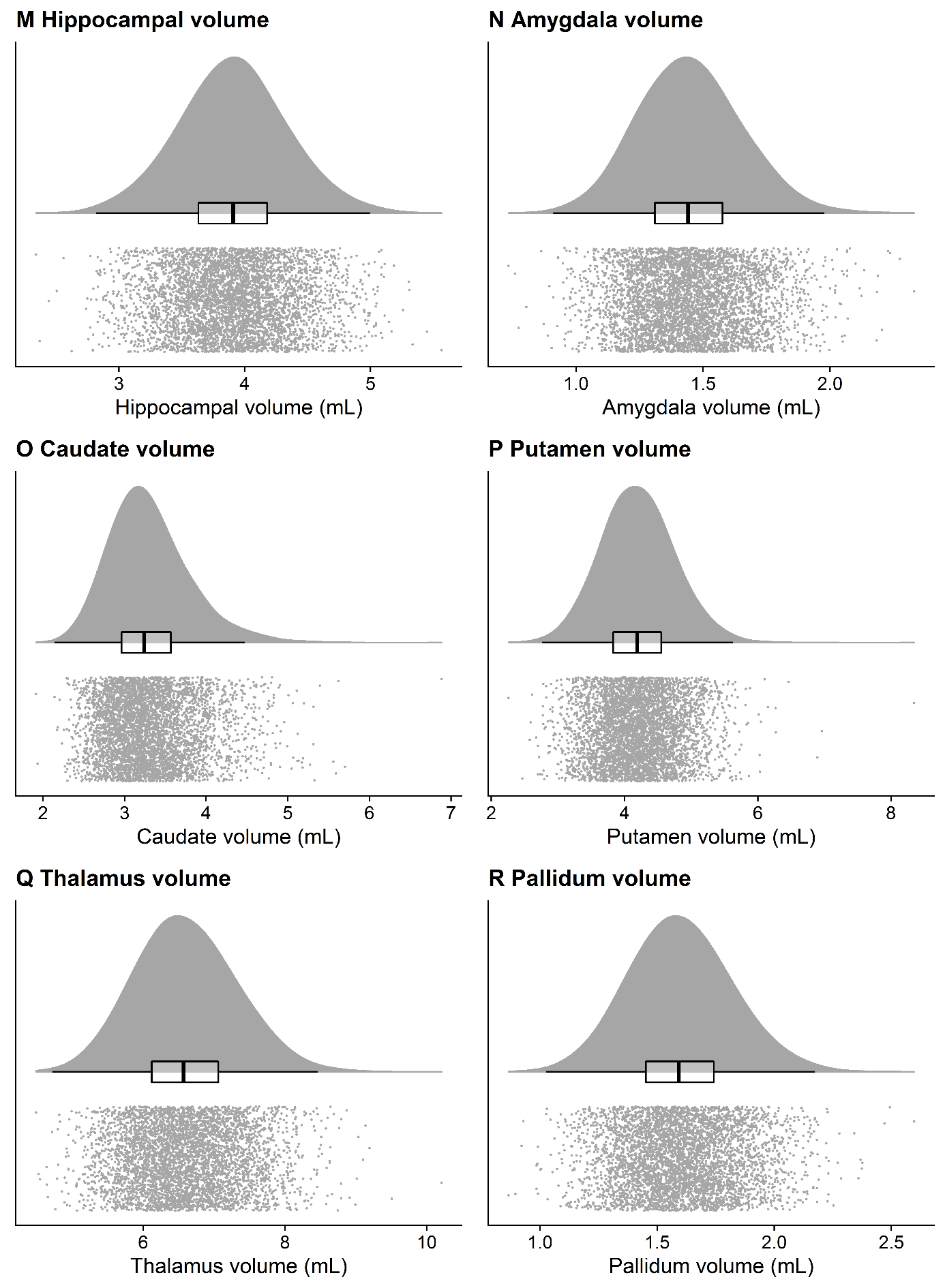
**

**
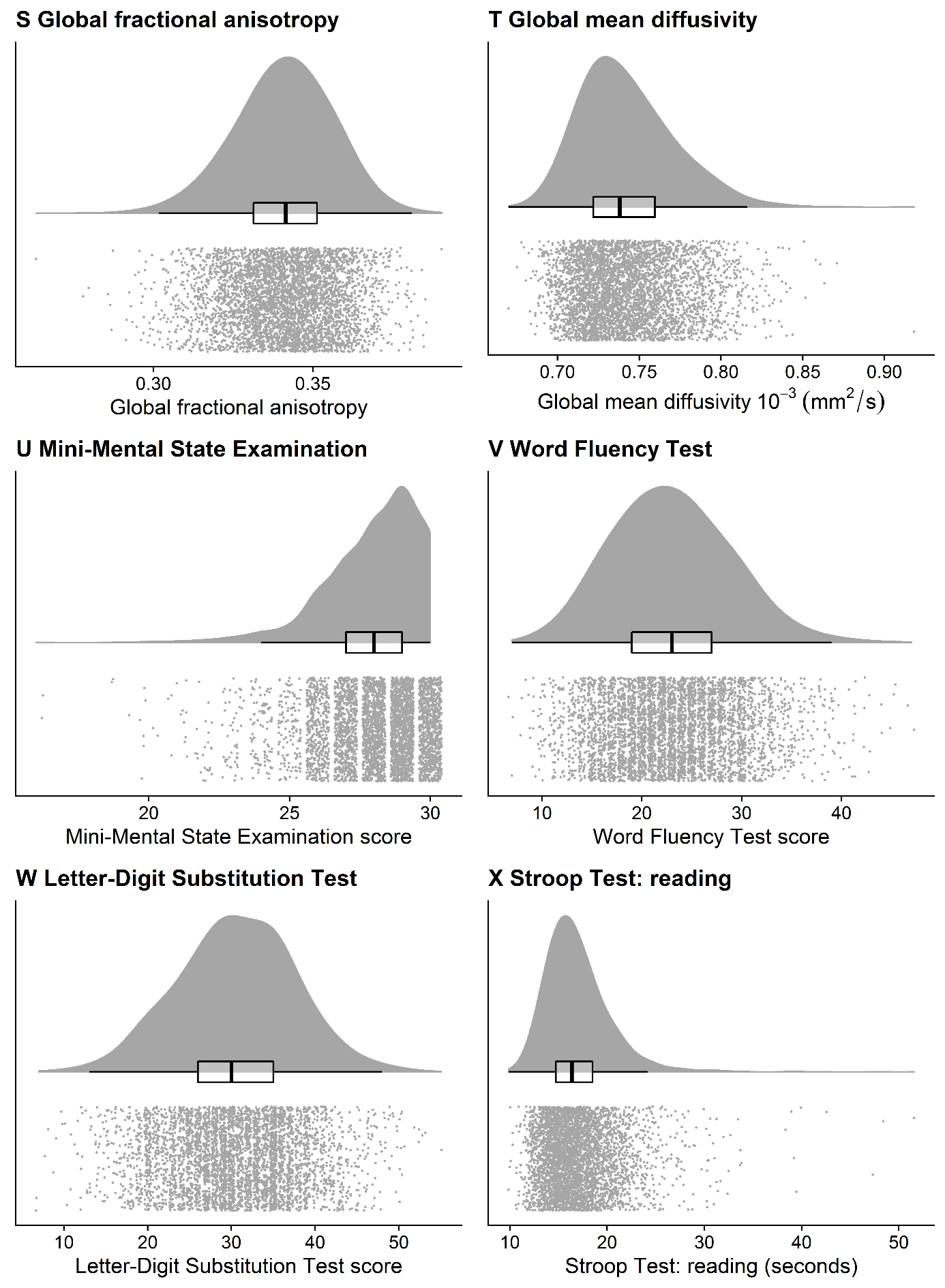
**

**
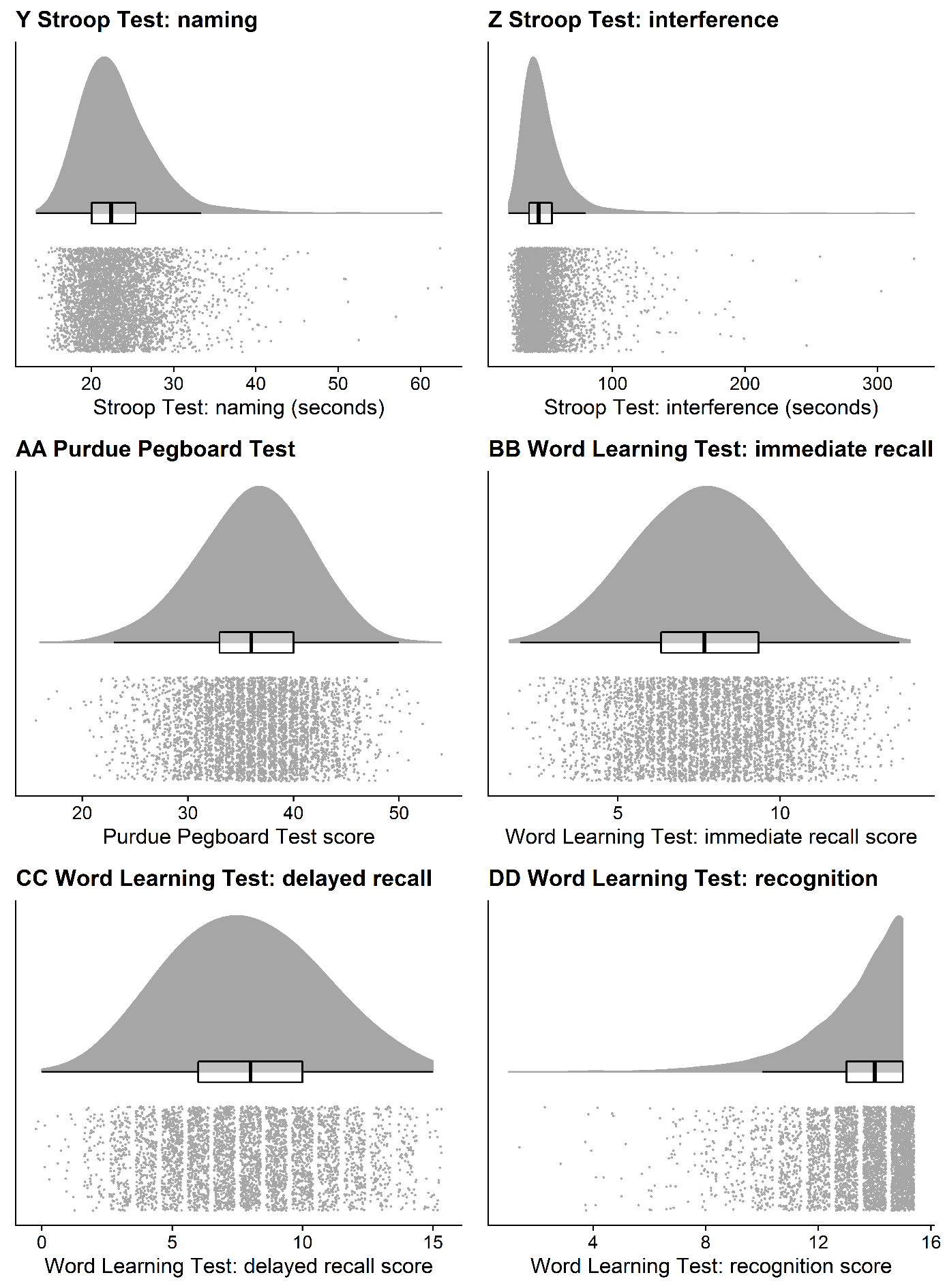
**

**
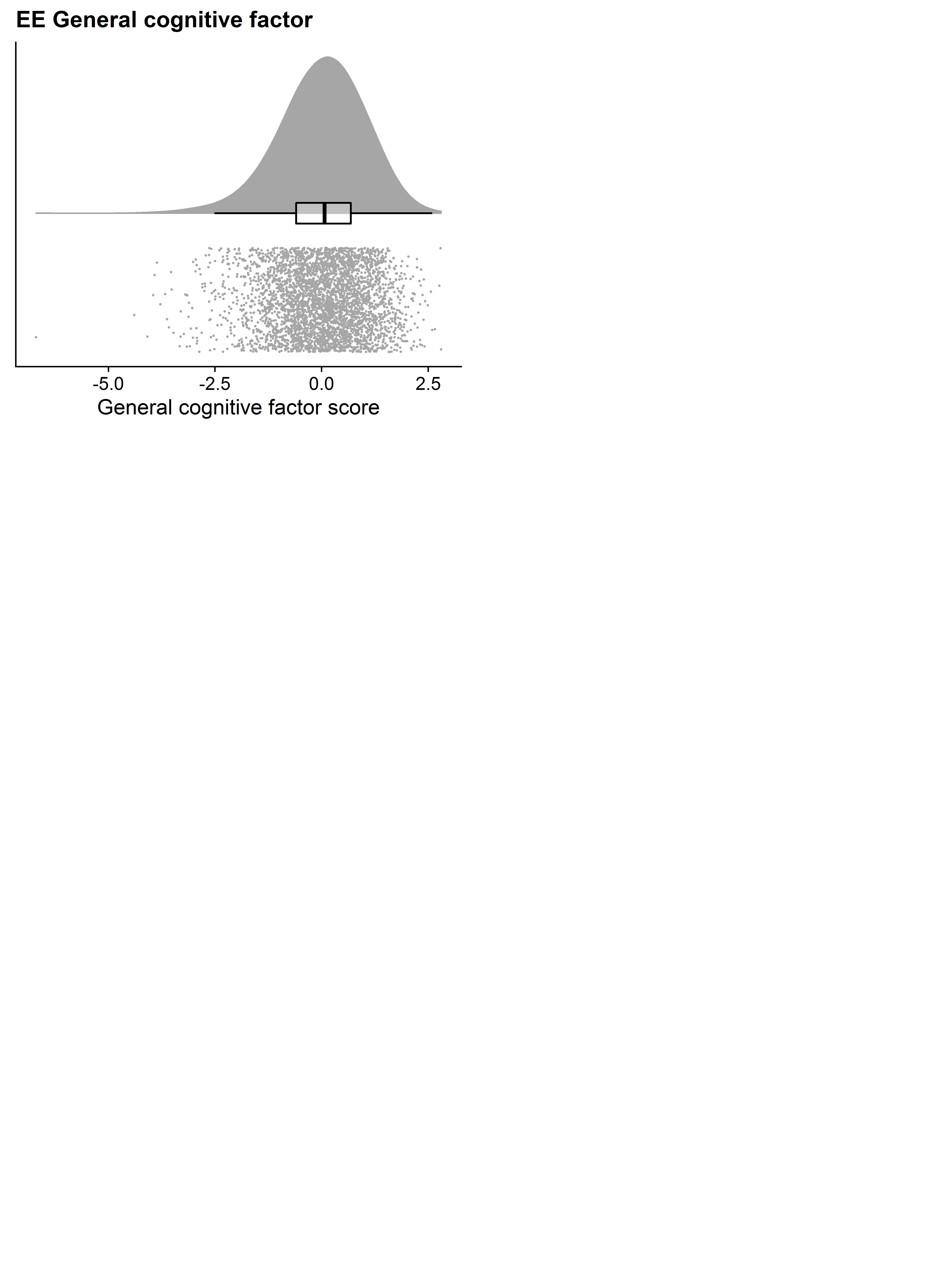
**

**Supplementary Figure 1** Raincloud plots for continous determinants and covariates that were used in the different models. The cloud represents the distribution of the data and the rain (grey dots) shows the jittered raw data. The boxplot shows the median and the interquartile ranges.

CES-D, Center for Epidemiological Studies Depression Scale.

**Supplementary Table 1 Details of used sequences.**

| **Sequence** | **Comment** | **Mode** | **Readout module** | **Time (min:sec)** | **TR/TE** | **TI (ms)** | **BW (kHz)** | **Flip angle (degrees)** | **Number of slices** | **Slice thickness (mm)** | **FOV (cm^2^)** | **Matrix** |
| --- | --- | --- | --- | --- | --- | --- | --- | --- | --- | --- | --- | --- |
| **PDw** |  | 2D | FSE | 6:09 | 12,300/17.3 |  | 17.86 | 90-180 | 90 | 1.6 | 25 | 416 x 256 |
| **T1w** |  | 3D | GRE | 6:24 | 13.8/2.8 | 400 | 12.5 | 20 | 96 (192) | 1.6 (0.8) | 25 | 416 x 256 |
| **FLAIR** |  | 2D | FSE | 6:25 | 8000/120 | 2000 | 31.25 | 90-180 | 64 | 2.5 | 25 | 320 x 224 |
| **DTI** | 25 directions; b = 1000 mm^2^/s, b_0_ NEX = 3 | 2D | EPI | 3:44 | 8000/74.6 |  | 250 | 90-180 | 39 | 3.5 | 21 | 64 x 96 |
| **T2*w** |  | 3D | GRE | 5:55 | 45/31 |  | 14.71 | 13 | 96 (192) | 1.6 (0.8) | 25 | 320 x 224 |

This Table has been obtained from ‘Ikram et al. The Rotterdam Scan Study: design update 2016 and main findings, 2015: European Journal of Epidemiology’.^1^

BW, bandwidth; DTI, diffusion tensor imaging; EPI, echo-planar imaging; FLAIR, fluid-attenuated inversion recovery; FOV, field of view; GRE, gradient-recalled echo; FSE, fast spin echo; NEX, number of excitations; PDw, proton density-weighted; T1w, T1-weighted; T2*w, T2*- weighted; TE, echo time, TI, inversion time; TR, repetition time.

**Supplementary Table 2** Association between markers of cerebral small vessel disease and risk of cancer when limiting the follow-up time to two years after brain MRI.

| **MRI measurement** | **Cancer**  **(n/N = 107/4,622)** | |
| --- | --- | --- |
|  | HR (95% CI) | *P*-value |
| White matter hyperintensity volume, mL^a,b^ | 0.94 (0.78-1.13) | 0.50 |
| Microbleeds | 0.85 (0.53-1.37) | 0.51 |
| Lacunar infarcts | 1.65 (0.95-2.86) | 0.07 |

Hazard ratios are adjusted for sex and total intracranial volume, education, body mass index, hypertension, diabetes mellitus, smoking status, alcohol use, and CES-D sum score.

^a^ Expressed per standard deviation increase. ^b^ Transformed with a natural logarithm.

CES-D, Center for Epidemiological Studies Depression Scale; CI, confidence interval; HR, hazard ratio; MRI, magnetic resonance imaging; n, number of participants with incident cancer; N, number of participants.

**Supplementary Table 3** Association between brain tissue volumes and microstructural brain measurements and risk of cancer when limiting the follow-up time to two years after brain MRI.

| **MRI measurement^a^** | **Cancer**  **(n/N = 107/4,622)** | |  |
| --- | --- | --- | --- |
|  | HR (95% CI) | *P*-value |  |
| *Global brain tissue volume, mL* | | |  |
| Total brain volume | 0.63 (0.35-1.12) | 0.12 | |
| Gray matter | 0.76 (0.51-1.14) | 0.18 | |
| Normal appearing white matter | 0.91 (0.66-1.24) | 0.54 | |
| *Lobar brain tissue volume, mL* | | |  |
| Frontal | 0.79 (0.53-1.17) | 0.23 | |
| Parietal | 0.96 (0.66-1.38) | 0.81 | |
| Temporal | 0.94 (0.65-1.35) | 0.73 | |
| Occipital | 0.97 (0.74-1.26) | 0.82 | |
| *Subcortical structure volume, mL* | | |  |
| Hippocampus | 0.75 (0.58-0.98) | 0.04 | |
| Amygdala | 0.90 (0.69-1.18) | 0.46 | |
| Caudate | 0.95 (0.78-1.17) | 0.64 | |
| Putamen | 0.90 (0.72-1.14) | 0.40 | |
| Thalamus | 0.78 (0.57-1.07) | 0.13 | |
| Pallidum | 0.84 (0.66-1.07) | 0.15 | |
| *White matter microstructure^b^* | | |  |
| Global fractional anisotropy | 1.06 (0.85-1.33) | 0.59 | |
| Global mean diffusivity, 10^-3^ mm^2^/s | 0.97 (0.74-1.27) | 0.80 | |

Hazard ratios are adjusted for sex and total intracranial volume, education, body mass index, hypertension, diabetes mellitus, smoking status, alcohol use, and CES-D sum score. For gray matter volume additionally adjustment for total white matter volume. For white matter microstructure additional adjustment for normal appearing white matter volume, white matter hyperintensity volume, and phase encoding direction.

^a^ Expressed per standard deviation increase. ^b^ Fractional anisotropy and mean diffusivity were measured in 4,354 participants due to missing diffusion tensor imaging data.

CES-D, Center for Epidemiological Studies Depression Scale; CI, confidence interval; HR, hazard ratio; MRI, magnetic resonance imaging; n, number of participants with incident cancer; N, number of participants.

**Supplementary Table 4** Association between markers of cerebral small vessel disease and risk of cancer stratified by cancer type.

| **MRI measurement** | **Prostate cancer**  **(n=57)** | | **Breast cancer**  **(n=46)** | | **Colorectal cancer**  **(n=63)** | | **Lung cancer**  **(n=37)** | | **Metastasized cancer**  **(n=61)** | |
| --- | --- | --- | --- | --- | --- | --- | --- | --- | --- | --- |
|  | HR (95% CI) | *P*-value | HR (95% CI) | *P*-value | HR (95% CI) | *P*-value | HR (95% CI) | *P*-value | HR (95% CI) | *P*-value |
| White matter hyperintensity volume, mL^a,b^ | 1.09 (0.78-1.51) | 0.62 | 1.13 (0.83-1.53) | 0.45 | 0.93 (0.71-1.22) | 0.62 | 1.03 (0.80-1.32) | 0.81 | 1.02 (0.78-1.32) | 0.91 |
| Microbleeds | 0.73 (0.35-1.51) | 0.39 | 1.07 (0.49-2.33) | 0.87 | 0.88 (0.47-1.65) | 0.70 | 0.54 (0.22-1.34) | 0.19 | 0.69 (0.32-1.52) | 0.36 |
| Lacunar infarcts | 1.56 (0.69-3.53) | 0.28 | 1.55 (0.47-5.08) | 0.47 | 1.27 (0.54-3.02) | 0.59 | 2.07 (0.88-4.88) | 0.10 | 1.28 (0.49-3.35) | 0.61 |

Models are adjusted for sex, total intracranial volume, education, body mass index, hypertension, diabetes mellitus, smoking status, alcohol use, and CES-D sum score.

^a^ Expressed per standard deviation increase. ^b^ Transformed with a natural logarithm.

CES-D, Center for Epidemiological Studies Depression Scale; CI, confidence interval; HR, hazard ratio; MRI, magnetic resonance imaging; n, number of participants with incident cancer.

**Supplementary Table 5** Association between brain tissue volumes and microstructural brain measurements and risk of cancer stratified by cancer type.

| **MRI measurement^a^** | **Prostate cancer**  **(n=57)** | | **Breast cancer**  **(n=46)** | | **Colorectal cancer**  **(n=63)** | | **Lung cancer**  **(n=37)** | | **Metastasized cancer**  **(n=61)** | |
| --- | --- | --- | --- | --- | --- | --- | --- | --- | --- | --- |
|  | HR (95% CI) | *P*-value | HR (95% CI) | *P*-value | HR (95% CI) | *P*-value | HR (95% CI) | *P*-value | HR (95% CI) | *P*-value |
| *Lobar brain tissue volume, mL* | | | | | | | | | | |
| Frontal | 1.16 (0.68-1.98) | 0.59 | 1.38 (0.75-2.53) | 0.30 | 1.37 (0.83-2.28) | 0.22 | 0.45 (0.23-0.89) | 0.02 | 0.98 (0.58-1.66) | 0.94 |
| Parietal | 1.03 (0.62-1.69) | 0.92 | 1.72 (0.97-3.07) | 0.06 | 1.47 (0.91-2.36) | 0.11 | 0.41 (0.22-0.77) | 0.01 | 1.16 (0.71-1.90) | 0.55 |
| Temporal | 0.78 (0.48-1.26) | 0.31 | 1.41 (0.78-2.55) | 0.26 | 1.70 (1.06-2.72) | 0.03 | 0.55 (0.29-1.04) | 0.07 | 1.10 (0.68-1.80) | 0.69 |
| Occipital | 0.97 (0.69-1.36) | 0.84 | 1.14 (0.74-1.74) | 0.55 | 0.89 (0.64-1.26) | 0.52 | 0.80 (0.50-1.28) | 0.35 | 0.81 (0.58-1.15) | 0.25 |
| *Subcortical structure volume, mL* | | | | | | | | | | |
| Amygdala | 0.94 (0.67-1.33) | 0.73 | 0.98 (0.63-1.53) | 0.94 | 1.04 (0.73-1.47) | 0.85 | 1.12 (0.70-1.81) | 0.64 | 1.04 (0.72-1.50) | 0.84 |
| Caudate | 1.25 (0.93-1.68) | 0.14 | 0.91 (0.64-1.30) | 0.60 | 0.86 (0.65-1.15) | 0.31 | 1.24 (0.89-1.73) | 0.20 | 1.05 (0.79-1.40) | 0.73 |
| Putamen | 1.08 (0.77-1.51) | 0.65 | 0.85 (0.57-1.26) | 0.42 | 0.98 (0.72-1.33) | 0.88 | 0.65 (0.42-1.02) | 0.06 | 0.83 (0.59-1.16) | 0.27 |
| Thalamus | 1.34 (0.91-1.96) | 0.13 | 0.77 (0.46-1.27) | 0.30 | 0.90 (0.60-1.35) | 0.61 | 0.46 (0.25-0.83) | 0.01 | 0.94 (0.62-1.44) | 0.78 |
| Pallidum | 1.13 (0.83-1.55) | 0.43 | 0.82 (0.54-1.23) | 0.33 | 1.00 (0.73-1.37) | 0.98 | 0.77 (0.50-1.18) | 0.23 | 0.98 (0.71-1.36) | 0.92 |
| *White matter microstructure^b^* | | | | | | | | | | |
| Global fractional anisotropy | 0.91 (0.63-1.30) | 0.59 | 0.95 (0.66-1.35) | 0.77 | 0.96 (0.70-1.30) | 0.77 | 1.05 (0.70-1.58) | 0.81 | 1.20 (0.83-1.75) | 0.33 |
| Global mean diffusivity,  10^-3^ mm^2^/s | 1.07 (0.69-1.66) | 0.77 | 1.11 (0.73-1.70) | 0.61 | 1.00 (0.69-1.45) | 0.98 | 0.97 (0.59-1.59) | 0.91 | 0.97 (0.62-1.52) | 0.88 |

Models are adjusted for sex, total intracranial volume, education, body mass index, hypertension, diabetes mellitus, smoking status, alcohol use, and CES-D sum score. For white matter microstructure additional adjustment for normal appearing white matter volume, white matter hyperintensity volume, and phase encoding direction.

^a^ Expressed per standard deviation increase. ^b^ Fractional anisotropy and mean diffusivity were measured in less participants due to missing diffusion tensor imaging data. In these analyses, 40 participants were diagnosed with prostate cancer, 39 with breast cancer, 54 with colorectal cancer, and 29 with lung cancer. Of the participants with cancer, 37 had metastasized disease.

CES-D, Center for Epidemiological Studies Depression Scale; CI, confidence interval; HR, hazard ratio; MRI, magnetic resonance imaging; n, number of participants with incident cancer.

**Supplementary Table 6** Association between markers of cerebral small vessel disease and risk of cancer stratified by sex.

| **MRI measurement** | **Women**  **(n/N = 157/2,574)** | | **Men**  **(n/N = 196/2,048)** | |
| --- | --- | --- | --- | --- |
|  | HR (95% CI) | *P*-value | HR (95% CI) | *P*-value |
| White matter hyperintensity volume, mL^a,b^ | 1.01 (0.85-1.21) | 0.87 | 0.96 (0.83-1.12) | 0.62 |
| Microbleeds | 1.12 (0.76-1.66) | 0.56 | 0.88 (0.62-1.25) | 0.49 |
| Lacunar infarcts | 1.60 (0.88-2.92) | 0.12 | 1.20 (0.77-1.87) | 0.42 |

Models are adjusted for total intracranial volume, education, body mass index, hypertension, diabetes mellitus, smoking status, alcohol use, and CES-D sum score.

^a^ Expressed per standard deviation increase. ^b^ Transformed with a natural logarithm.

CES-D, Center for Epidemiological Studies Depression Scale; CI, confidence interval; HR, hazard ratio; MRI, magnetic resonance imaging; n, number of participants with incident cancer; N, number of participants.**Supplementary Table 7** Association between brain tissue volumes and microstructural brain measurements and risk of cancer stratified by sex.

| **MRI measurement^a^** | **Women**  **(n/N = 157/2,574)** | | **Men**  **(n/N = 196/2,048)** | |
| --- | --- | --- | --- | --- |
|  | HR (95% CI) | *P*-value | HR (95% CI) | *P*-value |
| *Global brain tissue volume, mL* | | | | |
| Total brain volume | 0.73 (0.44-1.22) | 0.23 | 0.87 (0.57-1.33) | 0.52 |
| Gray matter | 0.83 (0.58-1.18) | 0.30 | 1.04 (0.77-1.38) | 0.81 |
| Normal appearing white matter | 0.91 (0.68-1.22) | 0.53 | 0.86 (0.69-1.08) | 0.19 |
| *Lobar brain tissue volume, mL* | | | | |
| Frontal | 0.81 (0.58-1.13) | 0.22 | 1.04 (0.78-1.38) | 0.80 |
| Parietal | 1.00 (0.73-1.37) | 0.99 | 0.81 (0.62-1.05) | 0.11 |
| Temporal | 0.99 (0.72-1.38) | 0.97 | 0.91 (0.71-1.18) | 0.49 |
| Occipital | 0.97 (0.77-1.23) | 0.81 | 1.00 (0.84-1.20) | 0.97 |
| *Subcortical structure volume, mL* | | | | |
| Hippocampus | 0.91 (0.72-1.16) | 0.45 | 0.91 (0.72-1.16) | 0.11 |
| Amygdala | 1.07 (0.84-1.36) | 0.58 | 1.07 (0.84-1.36) | 0.64 |
| Caudate | 0.94 (0.78-1.14) | 0.54 | 0.94 (0.78-1.14) | 0.15 |
| Putamen | 0.83 (0.67-1.02) | 0.08 | 0.83 (0.67-1.02) | 0.82 |
| Thalamus | 0.77 (0.58-1.01) | 0.06 | 0.77 (0.58-1.01) | 0.37 |
| Pallidum | 0.88 (0.71-1.10) | 0.26 | 0.88 (0.71-1.10) | 0.67 |
| *White matter microstructure^b^* | | | | |
| Global fractional anisotropy | 0.88 (0.72-1.07) | 0.20 | 1.06 (0.89-1.27) | 0.53 |
| Global mean diffusivity,  10^-3^ mm^2^/s | 1.15 (0.90-1.46) | 0.26 | 0.91 (0.73-1.14) | 0.42 |

Models are adjusted for total intracranial volume, education, body mass index, hypertension, diabetes mellitus, smoking status, alcohol use, and CES-D sum score. For gray matter volume additionally adjustment for total white matter volume. For white matter microstructure additional adjustment for normal appearing white matter volume, white matter hyperintensity volume, and phase encoding direction.

^a^ Expressed per standard deviation increase. ^b^ Fractional anisotropy and mean diffusivity were measured in less participants due to missing diffusion tensor imaging data. In these analyses, 129 out of 2,426 women were diagnosed with cancer and 152 out of 1,928 men were diagnosed with cancer.

CES-D, Center for Epidemiological Studies Depression Scale; CI, confidence interval; HR, hazard ratio; MRI, magnetic resonance imaging; n, number of participants with incident cancer; N, number of participants.

**Supplementary Table 8** Association between cognitive function and risk of cancer.

| **Cognitive test** | **Cancer** | | |  |  |  |  |
| --- | --- | --- | --- | --- | --- | --- | --- |
|  | n/N | HR (95% CI) | *P*-value |  |  |  |  |
| Mini-Mental State Examination | 353/4,614 | 1.00 (0.94-1.07) | 0.95 |  |  |  |  |
| Word Fluency Test | 347/4,486 | 1.01 (0.99-1.03) | 0.47 |  |  |  |  |
| Letter-Digit Substitution Test | 347/4,496 | 1.01 (0.99-1.03) | 0.34 |  |  |  |  |
| Stroop Test^a^ | | | |  |  |  |  |
| Naming | 334/4,343 | 1.02 (0.99-1.05) | 0.28 |  |  |  |  |
| Reading | 333/4,342 | 1.00 (0.97-1.02) | 0.84 |  |  |  |  |
| Interference | 333/4,336 | 1.00 (1.00-1.01) | 0.58 |  |  |  |  |
| Purdue Pegboard Test | 326/4,326 | 0.99 (0.97-1.02) | 0.56 |  |  |  |  |
| Word Learning Test | | | |  |  |  |  |
| Immediate recall | 327/4,278 | 1.01 (0.96-1.07) | 0.64 |  |  |  |  |
| Delayed recall | 327/4,277 | 1.00 (0.96-1.04) | 0.85 |  |  |  |  |
| Recognition | 331/4,318 | 0.97 (0.92-1.02) | 0.25 |  |  |  |  |
| General cognitive factor | 298/3,927 | 1.03 (0.89-1.20) | 0.66 |  |  |  |  |
| Self-reported memory complaints | | | |  | |  |  |
| More problems remembering | 342/4,486 | 1.18 (0.95-1.47) | 0.14 |  |  |  |  |
| Forgetting (daily) pursuits | 342/4,486 | 0.99 (0.77-1.26) | 0.91 |  |  |  |  |
| Word-finding problems | 342/4,486 | 1.17 (0.92-1.48) | 0.21 |  |  |  |  |

Hazard ratios are adjusted for sex, education, body mass index, hypertension, diabetes mellitus, smoking status, alcohol use, and CES-D sum score.

^a^ Better performance corresponds to lower scores.

CES-D, Center for Epidemiological Studies Depression Scale; CI, confidence interval; HR, hazard ratio; n, number of participants with incident cancer; N, number of participants.
